# Attentional capture mediates the emergence and suppression of intrusive memories

**DOI:** 10.1016/j.isci.2022.105516

**Published:** 2022-11-05

**Authors:** Nicolas Legrand, Olivier Etard, Fausto Viader, Patrice Clochon, Franck Doidy, Francis Eustache, Pierre Gagnepain

**Affiliations:** 1 Normandie University, UNICAEN, PSL Research University, EPHE, INSERM, U1077, CHU de Caen, Neuropsychologie et Imagerie de la Mémoire Humaine, Centre Cyceron, Caen, France; 2 Normandie University, UNICAEN, INSERM, COMETE, CYCERON, CHU Caen, 14000 Caen, France

**Keywords:** Biological sciences, Neuroscience, Cognitive neuroscience

## Abstract

Intrusive memories hijack consciousness and their control may lead to forgetting. However, the contribution of reflexive attention to qualifying a memory signal as interfering is unknown. We used machine learning to decode the brain’s electrical activity and pinpoint the otherwise hidden emergence of intrusive memories reported during a memory suppression task. Importantly, the algorithm was trained on an independent attentional model of visual activity, mimicking either the abrupt and interfering appearance of visual scenes into conscious awareness or their deliberate exploration. Intrusion of memories into conscious awareness were decoded above chance. The decoding accuracy increased when the algorithm was trained using a model of reflexive attention. Conscious detection of intrusive activity decoded from the brain signal was central to the future silencing of suppressed memories and later forgetting. Unwanted memories require the reflexive orienting of attention and access to consciousness to be suppressed effectively by inhibitory control.

## Highlights

• Emergence of intrusive images during memory suppression is decoded from the EEG signal
• Intrusive activity was detected using a perceptual model of reflexive attention
• A common neural system mediates the mental capture of memory and external activity
• Consciousness of intrusive activity during control predicted forgetting

## Graphical abstract

**Figure:**
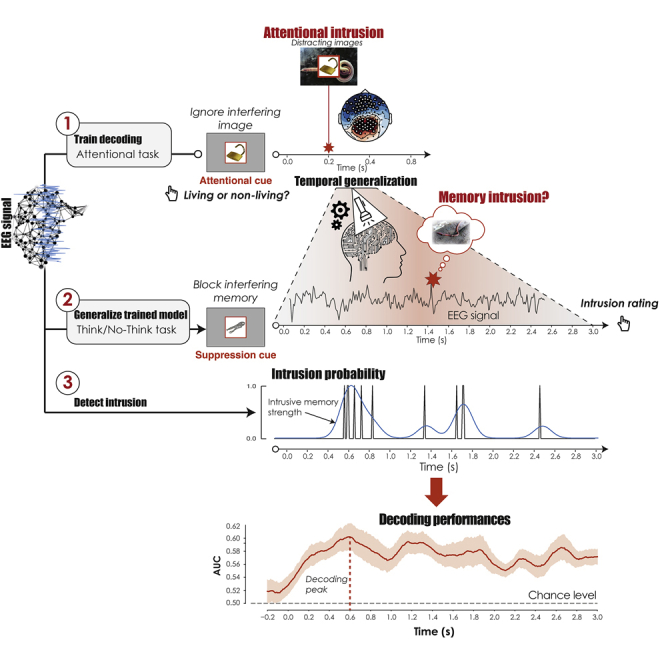

## Introduction

Distressing episodes of our life sometimes hijack our current focus of attention, disrupting cognitive or emotional goals.^1^ When these sensory images abruptly and involuntarily penetrate our consciousness, the brain can prevent their retrieval using inhibitory control.^2^^,^^3^^,^^4^ This process often results in a form of active forgetting, reducing the long-term accessibility of unwanted memory traces.^5^^,^^6^ Adaptive forgetting is central to emotion regulation,^7^^,^^8^ and its disruption can trigger or precipitate psychiatric disorders.^9^^,^^10^^,^^11^

The presence of an interfering signal indicating a memory’s irrelevance or intrusiveness is central to the premise of adaptive forgetting.^5^^,^^6^ However, the successful inhibition of interfering memories has mostly been observed indirectly and inferred a posteriori from subjective behavioral reports.^2^^,^^3^^,^^4^ The temporal dynamics of the inner memory signal triggering inhibition and indicating a memory’s irrelevance or intrusiveness, together with the role of attention in qualifying such activity as interfering, have never been precisely scrutinized. Up to now, the decoding of fMRI signals has consisted in tracking memory reactivation using perceptual templates of memory activity, in order to explore how memory inhibition relates to forgetting.^12^^,^^13^^,^^14^ In the present study, we decoded recordings of the brain’s electrical activity in order to study the temporal dynamics of the emergence of unwanted visual images in participants’ minds. Importantly, the classification of memories as intrusive was not based solely on the perceptual similarity between encoding and retrieval. Instead, we tried to characterize the oscillatory features that specifically reflected the redirection of attention toward intrusive elements liable to penetrate consciousness. To extract these features, we used a visual attention task designed to isolate the mental capture of intrusive images during active forgetting.

Visual attention can be voluntarily directed to a location or captured reflexively when sensory stimuli unexpectedly appear in the peripheral field.^15^ Voluntarily directed visual attention is endogenous and its control is triggered by symbolic indicators, whereas reflexive orienting of attention is exogenous and occurs automatically in response to salient stimuli in the visual field.^16^ These two distinct mechanisms interact, and the voluntary direction of attention in response to symbolic central cues can be interrupted by reflexive orienting of attention to peripheral stimuli, particularly when these are salient or unexpected.^17^^,^^18^ Interestingly, it seems that the attentional capture of memory-based representations is functionally similar to focusing attention on surrounding elements,^19^^,^^20^ suggesting that the reflexive orienting of attention toward intrusive memories may be partially captured by mechanisms involved in the visual detection of salient stimuli.

Moreover, accessing conscious content during memory reactivation^21^^,^^22^ or mental imagery^23^ implies the rapid reinstatement of early sensory processing in the occipitotemporal cortex, which is engaged during perception. The ventral parietal cortex, a region strongly associated with bottom-up attentional capture,^24^^,^^25^ is also engaged during remembering.^26^ Although the local processing of perceptual and memory content is segregated in this region,^27^^,^^28^ pattern-information analysis has been used to track memory reactivation in the ventral parietal cortex of visual categories relevant to task goals.^29^ Building on these close similarities between memory retrieval and the visual experience of external events, recent studies have undertaken multivariate decoding of electrophysiological signals to explore the reactivation of memory traces.^22^^,^^30^^,^^31^^,^^32^^,^^33^^,^^34^ Taken together, these studies suggest that the brain signal triggered by external attentional capture can be used to decode the emergence of intrusive memories and internal reflexive orienting mechanisms.

**Figure 1:**
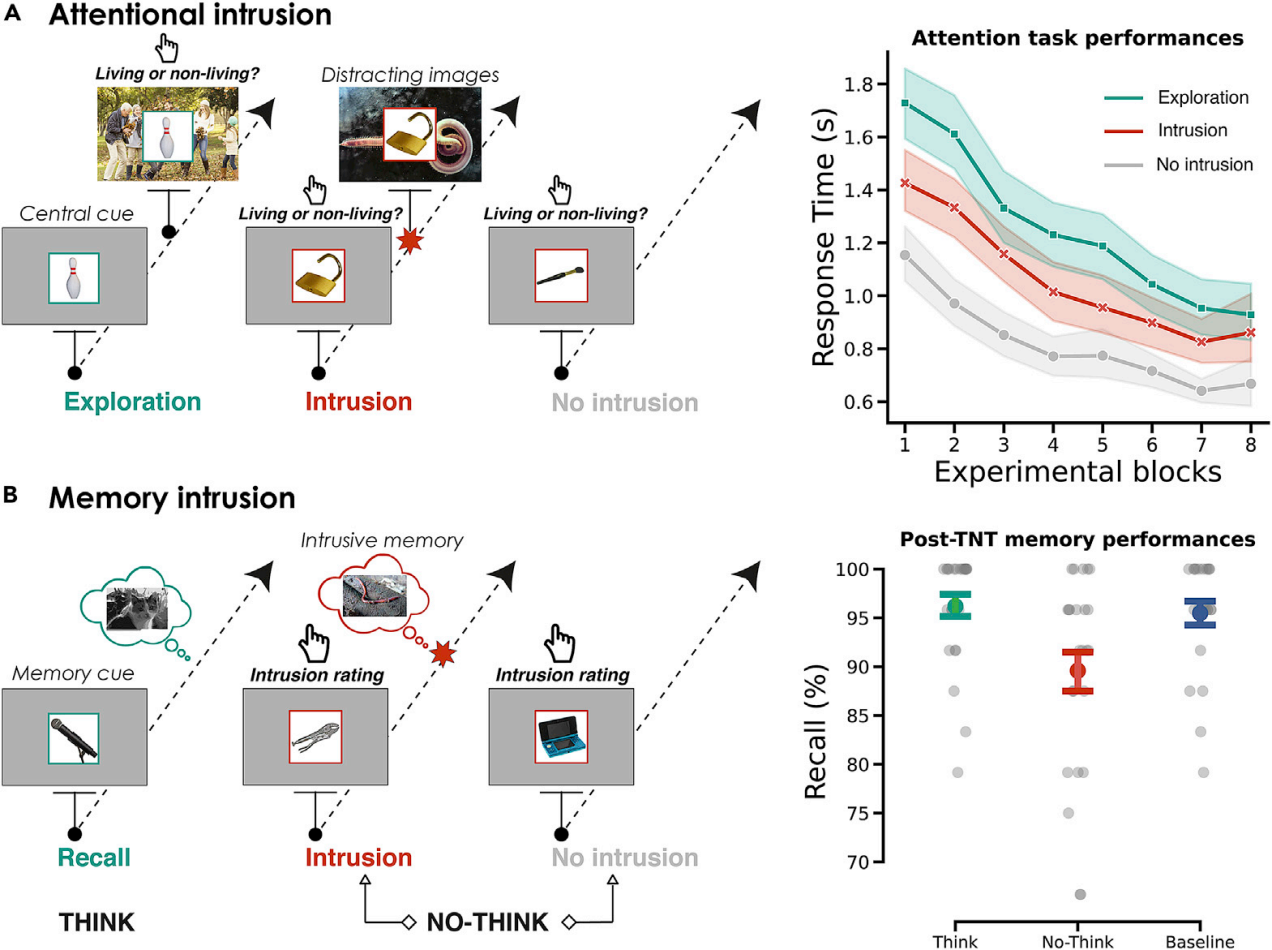
Experimental design and behavioral results (A) [left panel] Participants were instructed to categorize visual stimuli as fast as possible. Each trial started with the picture of an object displayed in the center of the screen, in either a green or a red box. A larger image appeared in the background 200 ms after the trial started in the exploration (green trials) and intrusion conditions (red trials), but not in the non-intrusion condition (red trials). For green trials, participants were instructed to explore this image and indicate whether living beings were present in the picture or not. For red trials, participants were instructed to stay focused on the central object and indicate whether the depicted image corresponded to a living or non-living thing. As for No-Think trials in the TNT task, participants were instructed to try their best to prevent the images in the background from entering their consciousness and to maintain their attention on the central object. If the background scene nevertheless penetrated their consciousness, participants were told to push it out of their mind and to fixate and concentrate on the object-cue. An image appeared in the background 200 ms after the onset of the central image for half the red trials (intrusion condition). In the other red trials, the target object was displayed without distracting scene (non-intrusion condition, depicted in gray). [right panel] Response times were recorded for the intrusion, non-intrusion, and exploration conditions. Overall, response times decreased across the eight experimental blocks, indicating a task-learning effect. However, for red trials, where participants had to focus on the central cue, the intrusion condition was associated with longer response times than the non-intrusion condition, indicating greater image interference. The shaded area represents the bootstrapped 95% CI. (B) [left panel] Memory suppression task (TNT paradigm). After the initial learning of the object-scene pairs (not shown here), Think and No-Think items were displayed in green or red boxes in the TNT phase (see main text for detailed instruction). [right panel] Same-probe recall of associated scenes after the TNT procedure. Memory performances were poorer for No-Think cues than for Think and baseline cues. Error bars represent bootstrapped 95% CIs.

To examine this question, we used multivariate decoding of EEG recordings to track memory intrusion during the Think/No-Think (TNT) task,^35^ based on bottom-up capture during an attention task (see Figure 1). The TNT paradigm is designed to induce intrusive memories triggered by a cue whose long-term accessibility is reduced after several suppression attempts. After the initial learning of object-scene pairs, cues are displayed in either a green or a red box during the TNT phase. For green cues, participants are told to generate a mental image of the associated scene that is as detailed as possible. For red cues, participants are told that it is vital to keep their minds blank and prevent the previously associated scene from coming to mind, without replacing the scene with any other thoughts or mental images. Participants are told that they should fixate and concentrate on the object-cue without looking away. If the scene nevertheless comes to mind, they are asked to push it out of their mind. After the offset of each TNT trial cue, participants report the extent to which the associated scene entered their awareness, making it possible to identify the No-Think trials that triggered intrusions.^3^^,^^4^ Memory performances are generally poorer for No-Think cues than for Think and baseline cues,^11^ although some studies have failed to replicate this effect.^36^ Baseline pairs are learned during the initial encoding phase, but are not presented in the TNT phase. These pairs make it possible to assess the effects of retrieval and suppression on the retention of Think and No-Think items, compared with similarly old pairs. Previous studies using the TNT paradigm in combination with fMRI have shown that attempts to control such intrusions are characterized by the downregulation of hippocampal activity, initiated by the right dorsolateral prefrontal cortex.^2^^,^^3^^,^^4^ The negative top-down inhibitory signal increased when a counter-intentional intrusion emerged into a person’s awareness and predicted later forgetting.^2^ This targeted top-down suppression can extend to the amygdala^3^ and visual cortex,^37^ presumably making suppressed memories less vivid and distressing over time. These findings suggest that the capture of the onset of an unwanted trace in consciousness, followed by its exclusion via inhibitory control, is fundamental to the success of suppression-induced forgetting^38^ (SIF), in addition to the disruption of hippocampal and memory processing in a systemic and untargeted fashion^39^ or pre-recollection,^38^ which may also lead to forgetting.

The neurobiological bases of SIF remain largely unknown, but seem to involve the mobilization of the GABAergic system which may increase the tonic inhibition of the principal cells in the hippocampus.^40^ Dampening the reactivation of the memory trace to a moderate level may facilitate the long-term depression of synaptic connections,^12^ possibly altering cellular consolidation. Alternatively, GABAergic inhibition may increase the silencing of the memory trace through the inhibitory engrams that mirror excitatory connections.^41^ Other non-inhibitory accounts suggest that SIF is related to the interference caused by the retrieval of distracting thoughts or memories that compete with the memory paired with the cue.^42^ Given the well-known effect of interference on consolidation mechanisms, interference accounts may also explain forgetting without recourse to inhibitory control (see discussion for further exploration of this point).

Higher temporal resolution of the brain signature of transient and unpredicted intrusive memory reactivation can be obtained with magneto- or electroencephalography (M/EEG). Previous studies have relied on event-related potentials (ERPs)^43^^,^^44^^,^^45^^,^^46^ or time-frequency representations^47^^,^^48^^,^^49^ to characterize the oscillatory signature of inhibitory control.^50^ However, these studies did not track the subjective reports associated with memory intrusion, leaving the alleged role of an interference signal in adaptive forgetting untested. Moreover, including these behavioral reports is not sufficient, given that intrusive mental images are unpredictable and the precise timing of their appearance unknown, precluding inferences on the neural signature of individual intrusive mental images.

To circumvent this problem, we trained a classifier to detect intrusive memories, based on an attention task that was designed to closely reproduce the abrupt intrusion of a visual scene into awareness. This intrusion interfered with an ongoing concurrent task performed on a central cue (i.e., a living/non-living classification decision task; see Figure 1A). During this task, attention was voluntarily directed to a central object cue in a green or red box (as in the TNT). A green box instructed participants to redirect their attentional focus to a large scene that appeared in the background 200 ms (ms) after the start of the trial (exploration condition mimicking the voluntary redeployment of attention to a scene, as in the Think condition). As with No-Think trials during the TNT task, a red box instructed participants to maintain their attention on the central object. In half of these trials, an intrusive image appeared in the background 200 ms after the start of the trial (intrusion condition involving reflexive attention), and participants were instructed to try their best to block the images from entering their consciousness. If the background scene did penetrate their consciousness, participants were told to push it out of their mind and to fixate and concentrate on the object-cue. In the other red trials, no intrusive image appeared (no-intrusion condition).

Using the EEG signal from this attention task, we then trained a classifier to predict the reactivation of intrusive mental content in the EEG signal of the memory suppression task. The intrusion ratings provided by the participant at the end of each trial were used to estimate the true occurrence/absence of intrusions and evaluate the accuracy of the model’s prediction across the duration of the presentation of the suppression cue. A major difference between the attention and TNT tasks we administered is that the appearance of the intrusive image during the former did not depend on a subjective report. The great advantage of this distinction is that it ensured that the decoding of intrusive memory in consciousness depended on neural patterns that were not biased by cognitive access to the content of consciousness and the ability to provide a behavioral report of consciousness, which may be a non-necessary consequence of consciousness, according to some models.^51^^,^^52^ According to Koch et al. (2016), the term *consciousness* refers to a state in which content can be subjectively experienced through sensory-based neural activities. The neural correlate of consciousness can arise from early visual processing, and may not necessarily require a behavioral report.^53^^,^^54^ We, therefore, reasoned that we could accurately decode the raw EEG signal of the TNT task to pinpoint the occurrence of these intrusive visual patterns and thereby infer the moment in time when an intrusive memory entered awareness for a given trial, using a training task for the generalization decoding algorithm that does not require such a report. Furthermore, a fundamental idea associated with inhibitory control is that this mechanism countermands interfering activity that violates current goals, in order to adapt and reduce inappropriate responses (here, intrusive memories). It is not yet known, however, whether reflexive attention and attentional capture are needed to qualify an activity as interfering, or whether only some form of perceptual activity, not necessarily intrusive, is required. We hypothesized that although perceptual activity may be sufficient to decode memory activities, it may not be sufficient to capture the intrusive nature (i.e., nonintentional retrieval) of suppressed memories (assumed here to reflect the reflexive orienting of attention) or to ascertain how intrusive content can be regulated and forgotten. To test this, we compared a decoding scenario in which attention was reflexively oriented by an abrupt change in the visual background scene (intrusion condition) with a situation in which attention was voluntarily directed to the perceptual activity related to scene processing, but without any breach in expectation or reflexive orienting (exploration condition). We also evaluated the temporal dynamics of the decoded intrusive mental event and assessed how intrusive memories might transition to non-intrusive states and how the impact of repeated control might modulate these dynamics. The frequency of memory intrusions is known to gradually decrease over suppression sessions - an effect associated with the magnitude of subsequent forgetting.^4^ Here, we tested whether this also applied to our decoded mental events and extended their characterization to the temporal domain of the suppression cue.

## Results

### Behavioral results

#### Validation of attentional model of intrusion - Intrusive images interfere with categorization

First, we wanted to test the effect of the attentional capture induced by the appearance of an unexpected scene in the background during the semantic categorization task performed on the central image. We predicted that this would increase the response time, owing to the need for participants to inhibit irrelevant stimuli and redirect attentional focus while performing image categorization. We averaged response times for each of the attentional conditions (exploration, intrusion, non-intrusion) and each of the eight experimental blocks (see Figure 1A). We only report results for the trials that survived artifact rejection during the EEG preprocessing (see STAR Methods). A Block x Condition ANOVA on the averaged response times revealed significant main effects of condition, *F*_(2, 46)_ = 99.38, $\eta_{p}^{2}$= 0.81, p < 0.001, and block, F_(7, 161)_ = 98.72, $\eta_{p}^{2}$= 0.81, p < 0.001, and an interaction between the two, *F*_(14, 322)_ = 7.59, $\eta_{p}^{2}$= 0.24, p < 0.001. Planned comparisons revealed that response times were shorter for the non-intrusion condition than for both the intrusion, *t*_(23)_ = −10.61, *d* = −1.16, p < 0.001, and exploration, *t*_(23)_ = −13.09, *d* = −1.86, p < 0.001, conditions. The longer response time in the intrusion condition, compared with a situation with no perceptual interference, suggests that the abrupt onset of an interfering background image effectively induced a reflexive orienting process that slowed down reaction times to the attended central object. We interpreted this result as the corroboration of our attentional intrusion model.

#### Effect of inhibitory control on memory performances

Intrusion ratings were used to isolate reminders associated with intrusive memories and to quantify their occurrence in a binary fashion. For each repetition of an No-Think trial (eight in total), we averaged these binary intrusion reports across all items to compute the temporal dynamics of intrusion proportion. The TNT block ANOVA revealed that the inhibitory control of memory recall was characterized by a gradual decrease in the proportion of intrusions during the TNT task (*F*_(7, 154)_ = 10.23, $\eta_{p}^{2}$ = 0.31, p < 0.001). The frequency of memory intrusions is known to gradually decrease over suppression sessions, as participants exert inhibitory control - an effect associated with the magnitude of subsequent forgetting.^4^ In this context, the proportion of intrusions for each repetition of the suppression cue [*n*_intrusion_/(*n*_intrusion_ + *n*_non-intrusion_)] serves to estimate the resulting slope, using Pearson correlation coefficients that describe the regulation of intrusion across suppression attempts. Here, we reproduced this finding and found that the steeper the intrusion slope, the greater the SIF. It should, however, be noted that this relationship was reliably significant with respect to the bootstrapped confidence interval (CI), but not significant with respect to its associated p value (*R*_Spearman_ = −0.33; 90% CI = [-0.56, −0.07]; p = 0.057).

We then tested the presence of a successful inhibitory control effect by comparing final recall performances in the Think, No-Think, and baseline conditions (see Figure 1B). We controlled for the presence of false recall, using audio recordings of participants’ descriptions of the recalled scene. A repeated-measures ANOVA on the percentage of correct recall during the final test, with condition as a factor, revealed a significant difference between conditions, *F*_(2.46)_ = 15.97, $\eta_{p}^{2}$= 0.40, p < 0.001. Critically, while no difference was found between Think and baseline items, *t*_(23)_ = 0.89, p > 0.5, *d* = 0.11, a planned comparison between No-Think and baseline items revealed that participants recalled significantly fewer items in the No-Think condition, *t*_(23)_ = −3.67, p = 0.003, *d* = 0.71. This result confirmed that the TNT procedure induced successful suppression of unwanted memories.

### EEG and intrusion decoding

#### Decoding the interference of intrusive images during the attention task

**Figure 2:**
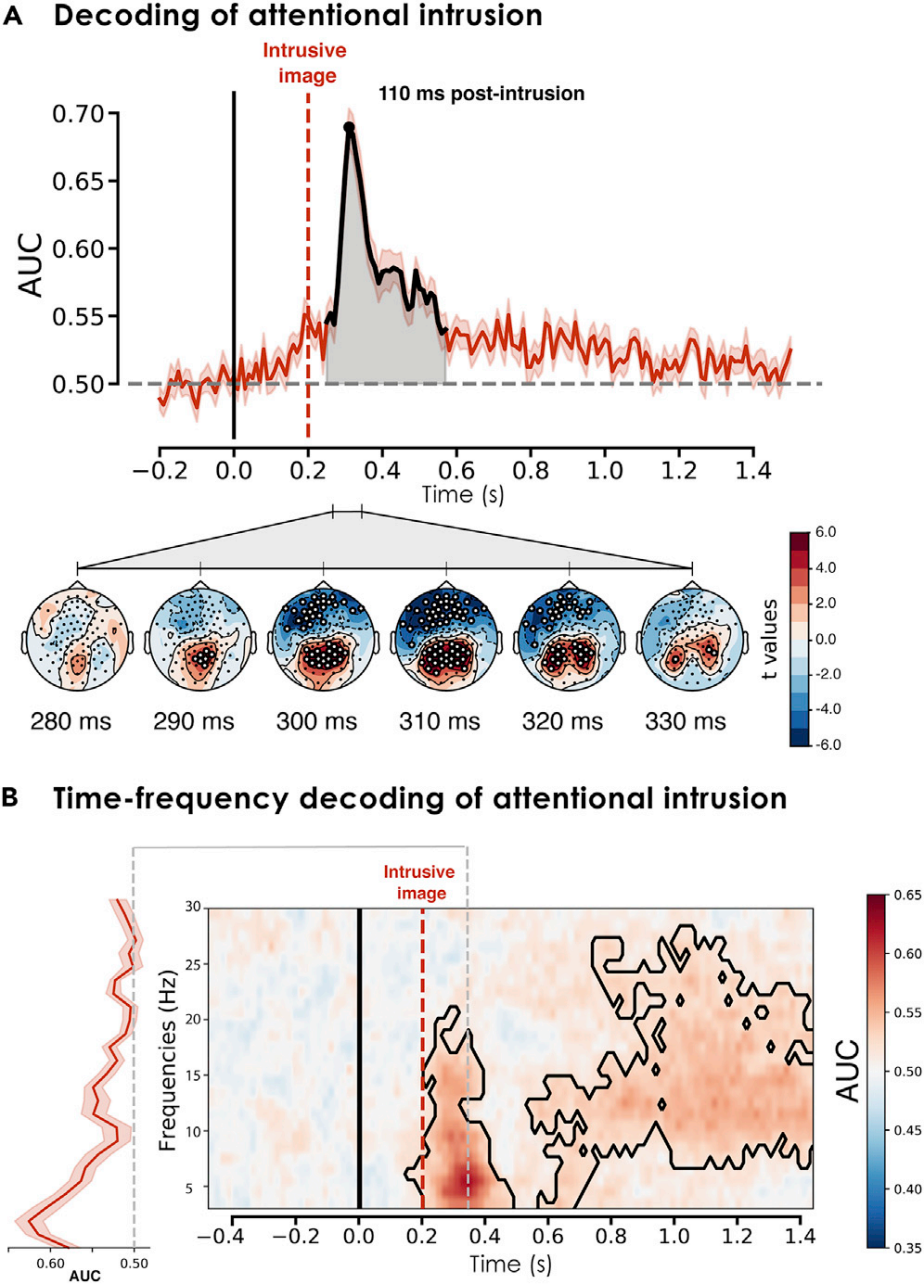
Decoding performances during the attention task (A) Random forest classifiers had performances significantly higher than chance level 250-580 ms following the onset of the initial central target image (the intrusive background image appeared at 200 ms and is marked here with a red dashed line; see Figure 1A for details of attention task). The topographic maps in the bottom part of the figure represent the contrast between the intrusion and non-intrusion conditions. Higher decoding scores were associated with significantly higher electrical activity over occipital electrodes, coupled with a significant reduction in activity over frontal electrodes. (B) Decoding performances over time and frequencies during the attention task. To pinpoint the contributions of different frequency bands supporting higher classification accuracy, we applied the decoding approach to all frequency bands between 3 and 30 Hz. The central panel shows the AUC for each time and frequency point. The black lines highlight the contours of the significant clusters revealed by the one-sample cluster permutation test (20,000 permutations). We found an early significant increase in decoding performance centered around the theta frequency range, as well as a late (700-1500 ms) increase preferentially centered around the beta frequency range. The gray dashed line indicates the time peak of maximum decoding accuracy 160 ms after the appearance of the intrusive image on the screen. The left subpanel shows the decoding AUC across frequencies at the highest decoding score timepoint. Theta frequency (3-7 Hz) encoded more information (maximum AUC reached at 5 Hz).

Based on the previous observation that the appearance of an intrusive image 200 ms after the onset of the central object interfered with the ongoing categorization task, we first sought to decode the EEG signal to pinpoint the signature of this attentional capture. We used the preprocessed voltage EEG signal from 102 electrodes of interest (see STAR Methods) and applied a random forest classifier at each timepoint to detect the presence or absence of a distracting background image (intrusion vs. non-intrusion condition). We trained and tested a model (50 trees) at each timepoint (1 sample/10 ms) on either side (−200 ms-1500 ms) of the onset of the central image (i.e., appearance of distracting image on screen). These classifiers were embedded in an 8-fold cross-validation framework, and performance was measured using the mean area under the curve (AUC) at each timepoint for each participant. The performances of the classifiers over time are provided in Figure 2A. At the group level, we averaged the AUC scores and tested the difference from chance level (AUC = 0.5) at each timepoint, using the cluster-based permutation *t* test implemented in the permutation_cluster_1samp_test() function of the MNE Python package^101^, and controlling for the familywise error rate across multiple timepoints.^57^ This procedure revealed an early significant decoding time window 250-580 ms after the onset of the central target object (cluster p value <0.001), with maximum decoding accuracy (AUC = 0.69, *SD* = 0.07) occurring 110 ms after the appearance of the intrusive image (see Figure 2A).

It should be noted that random forest classifiers can only decode the existence of distinct patterns of activity across EEG sensors, and do not provide any specific information about the contributions of individual sensors or frequency bands. To increase the interpretability of our result, we also explored the spatial distribution of these EEG activities and compared the intrusion and non-intrusion conditions in the main window of interest. For each electrode, we averaged the signal across time samples surrounding the peak of decoding accuracy, corresponding to a 280-340 ms time window after the onset of the central object. We restricted our analysis to this interval, as it overlapped with the temporal cluster of maximum decoding performances. We then ran a one-sample *t* test (permutation_t_test function()) to reveal potential differences between the intrusion and non-intrusion conditions. The resulting *t* values and significant clusters are reported in the lower panel of Figure 2A. These revealed a significant increase in the EEG signal over occipital electrodes and a significant decrease over frontal electrodes when the intrusion condition was contrasted with the no-intrusion condition.

To describe the oscillatory signature of attentional capture, we also generalized our decoding approach to the frequency domain and provided the classifiers with filtered time series at different ranges between 3 and 30 Hz. The results are described in Figure 2B. The signal was filtered using Morlet wavelets, as implemented in MNE Python, resulting in 27 signals corresponding to frequencies between 3 and 30 Hz. At each timepoint, and for each frequency, we extracted the averaged AUC score using the same 8-fold cross-validation scheme. In the resulting time-frequency heatmap, we observed that the maximum classification performance for the early presence or absence of the intrusive image (i.e., 160 ms after the intrusive scene) depended on theta (3-7 Hz) frequencies. This early decoding also depended on alpha (8-12 Hz) and low beta (13-20 Hz) ranges (see Figure 2B).

#### Decoding the mental capture of intrusive memories during memory suppression using a model of attentional interference

**Figure 3:**
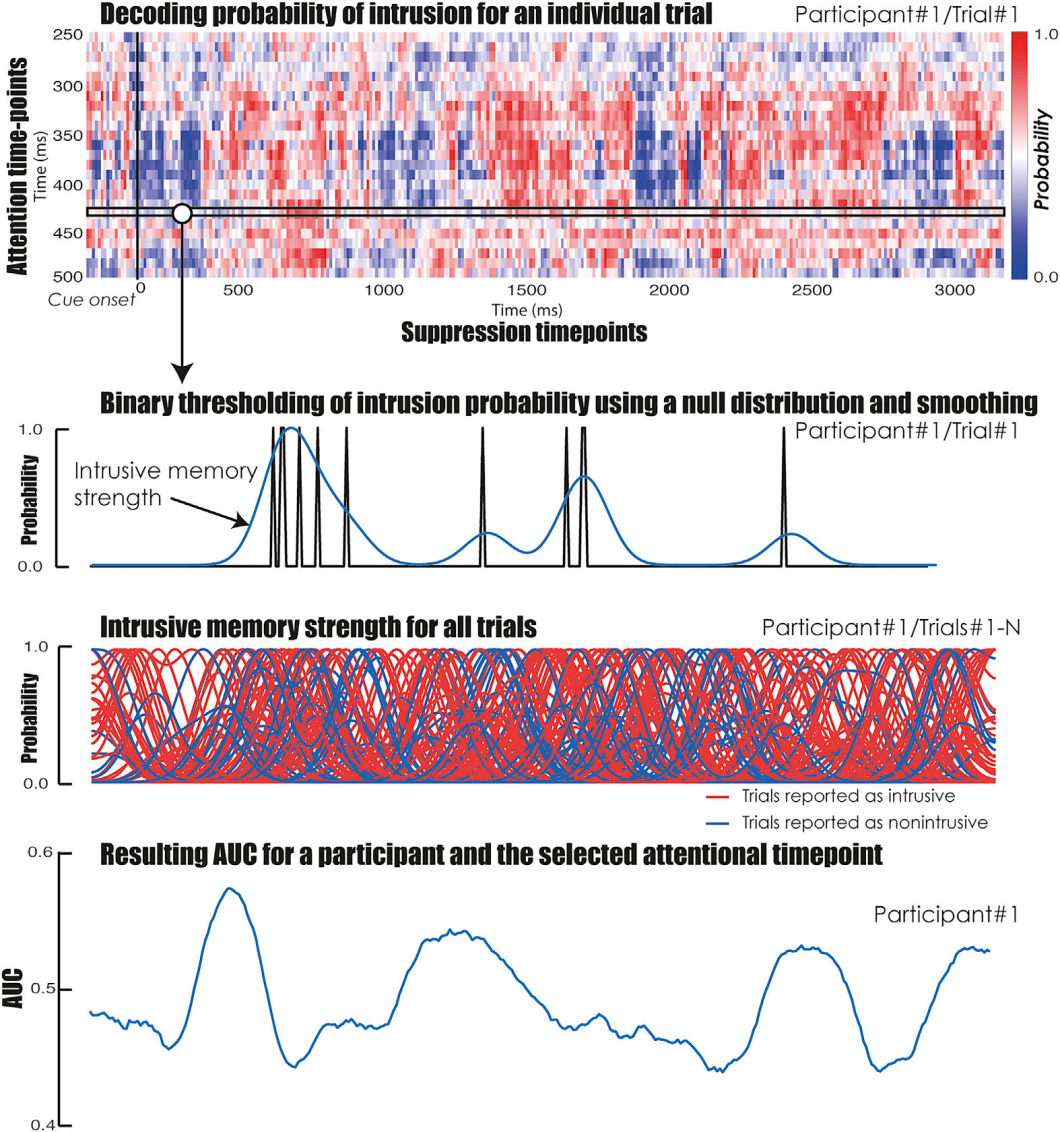
Decoding memory intrusion during the TNT task We predicted the probability of an intrusion during memory suppression (i.e., No-Think trial of the TNT phase). We used the classifiers trained on the EEG recording from the attention task, which captured visual and attentional interference processes associated with the appearance of the intrusive image (i.e., 250-500 ms post-cue onset). For each attentional timepoint (only one timepoint is illustrated here), we labeled peaks of probability above the 95th percentile of the null distribution as intrusions (see STAR Methods). The resulting binary vector was smoothed with a Gaussian kernel (with a full width at half maximum of 200 ms), producing a timecourse of mental event reactivation describing intrusive memory strength or probability across the suppression cue. The whole process was repeated across all No-Think trials, and we then computed the resulting AUC for each timepoint using the subjective TNT reports as labels, and the probability of an intrusion across trials as a vector of classifier predictions. We repeated the whole process for each attentional timepoint and selected for each participant the attentional timepoint with the maximum AUC. Follow-up analyses on the intrusive memory strength were also derived from the attentional timepoint with the maximum AUC.

**Figure 4:**
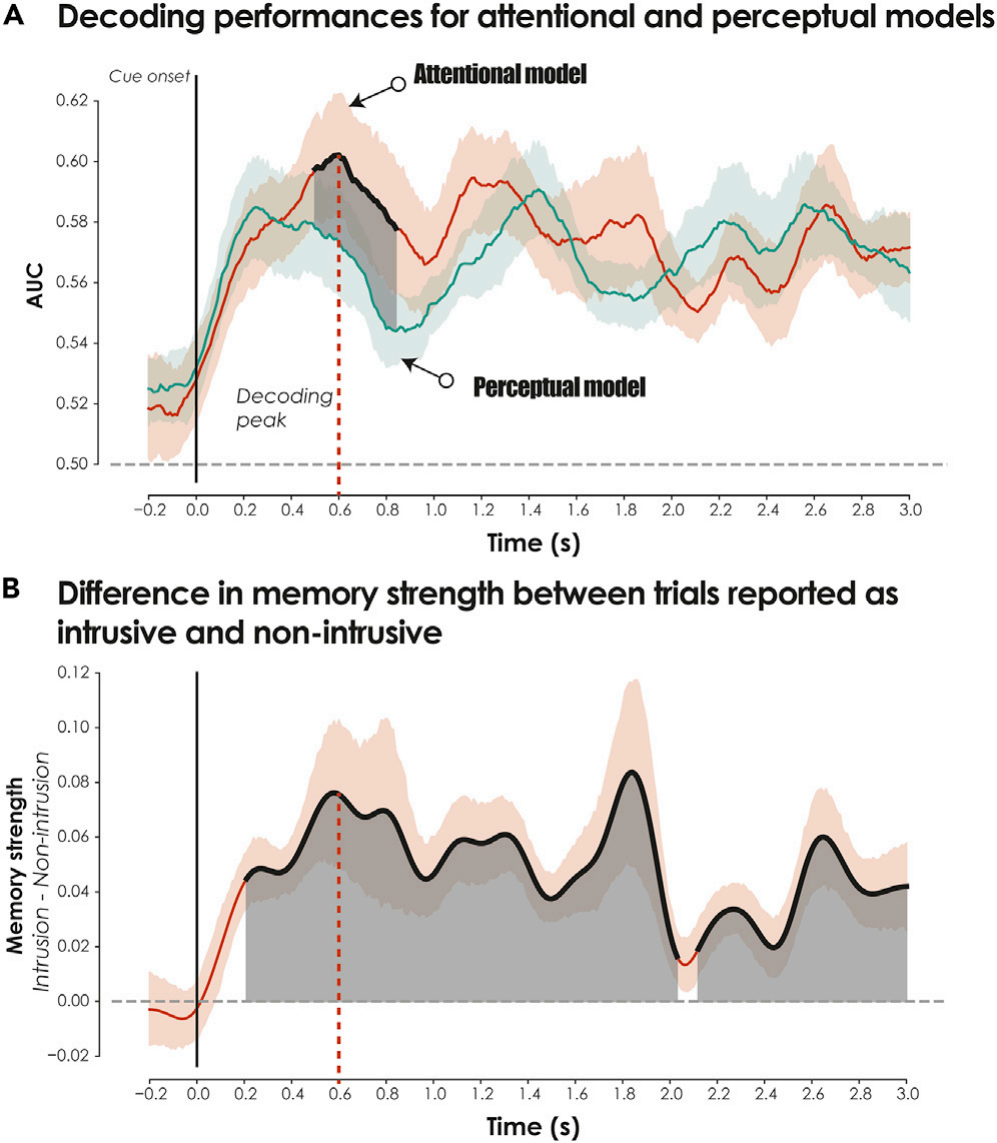
Decoding performance for intrusion reports during memory suppression (A) Performances of the attentional and perceptual classification models are reported here as AUCs. The attentional model (red line) detected intrusive memories using a classifier trained on reflexive attention (i.e., intrusion vs. non-intrusion during attention task; see Figure 1A). The perceptual model (green line) was trained to detect intrusive memories based on the voluntary perceptual processing of the scene (i.e., exploration vs. non-intrusion during attention task; see Figure 1A). The dark area represents the significant differences between these two classifiers, corrected for multiple comparisons across timepoints (p-corrected < 0.05). The dashed red line represents the decoding peak of the attentional model. (B) Analysis of intrusive memory strength (see Figure 3) confirmed that the increase in classification performances for the attentional model was characterized by an increase in intrusive mental events for trials reported as intrusive, compared with those labeled non-intrusive by participants (significant differences corrected for multiple timepoints represented by dark areas). Error bars represent bootstrapped 95% CIs.

After confirming that our model was able to detect the attentional capture of intrusive and distracting images, we tested its ability to decode intrusion reports during memory suppression. This approach has been described as the temporal generalization method, in which decoders trained at one timepoint are tested at another timepoint to infer the dynamics of mental representation.^55^ While the temporal generalization method often time-locks the trained and tested time series to the trial onset,^56^ we further generalized this approach by training and testing the decoder on two separate tasks, and by looking at the timecourse of the decoding probabilities for each trial, instead of the overall classification performances at each timepoint. For each participant and each trial, we first trained the random forest classifiers using the temporal activity of intrusion and non-intrusion from the attention task (i.e., attentional model), and tested their performances on decoding intrusive EEG patterns for every timepoint of the suppression cue during the TNT task (see Figure 3). Accordingly, the classifiers provided a probability of intrusion for every timepoint of the suppression cue, as well as every timepoint of the attention task used for training. We restricted our decoding approaches to the decoders trained between 250 ms and 500 ms after the presentation of the central object in the attention task (i.e., 50-300 ms after the appearance of the intrusive image). This procedure ensured that our model captured the neural activity associated with the most accurate decoding of the abrupt intrusion of an unexpected image into conscious awareness identified during the initial attentional decoding (see Figure 2A). To further increase the reliability of our decoding procedure, we estimated the null distribution of decoding probability by repeating the classification analysis 200 times with randomly permuted labels in the training dataset (attention task), as described by Linde-Domingo and colleagues (2019). We then used a threshold corresponding to the 95^th^ percentile of this null distribution to accept the prediction of an intrusion. This additional step reduced the odds of the prediction of an intrusive image being induced by noise in the EEG signal. The resulting binary map (corresponding to *N*_attention_ x *N*_TNT_ timepoints) for a given trial was then convolved with a Gaussian kernel (with a full width at half maximum of 200 ms), producing a timecourse of mental event reactivation describing the probability of an intrusion across No-Think trials. The label of each trial was based on the subjective reports provided by participants after suppression cues. For each timepoint of the *N*_attention_ x *N*_TNT_ matrix, we then computed the resulting AUC using the subjective TNT reports as labels and the probability of an intrusion across trials for this particular timepoint as a vector of classifier predictions. For each participant, we selected the attentional timepoint with the maximum AUC, producing a timecourse of the model classification performance across timepoints of the suppression cue. We performed a series of one-sample *t* tests at each timepoint of the TNT cue, to compare the AUC with chance level (i.e., 0.5), controlling for the familywise error rate across multiple timepoints using a cluster-based permutation *t* test.^57^ We observed that the maximum decoding accuracy of intrusion reports occurred 600 ms after the onset of the suppression (AUC = .602; *t* peak_(23)_ = 5.05, cluster p value <0.001; see Figure 4A).

#### Role of mental capture in decoding memory intrusiveness

To disentangle the role of reflexive orienting, we reapplied the previous decoding procedure, but this time training the attentional classifier using exploration versus non-intrusion trials to detect intrusion versus non-intrusion trials during the TNT task (i.e., perceptual model). Contrary to the intrusion condition, during which attention was reflexively oriented to the background by an abrupt change in the visual scene disrupting the processing of the central cue, no breach in expectation was involved during the exploration condition, in which attention is voluntarily directed to the scene. Subjective TNT reports were also decoded significantly above chance level in the exploration condition, with maximum decoding accuracy occurring 1450 ms after the onset of the suppression (AUC = 0.591; *t* peak_(23)_ = 6.04, cluster p value <0.001). Interestingly, however, an increase in AUC classification performance was observed for the attentional model compared with this perceptual model, in an early time window of 500-850 ms (*t* peak_(23)_ = 3.45, cluster p value <0.05; see Figure 4A).

We then sought to further characterize the temporal dynamics of intrusive mental events and intrusion control and confirm that the increase in classification performances for the attentional model was associated with an increase in intrusive memory strength during trials reported as intrusive. We, therefore, compared the timecourse of mental event reactivation describing the probability of an intrusion for No-Think trials reported as intrusive with those not labeled such by participants. We found a large and significant temporal cluster (cluster-based correction) of mental reactivation during intrusive trials, with an early peak of mental capture at 600 ms largely overlapping with the maximum decoding time window of the attentional classifier (see Figure 4B).

#### Temporal dynamics of intrusion control and adaptive forgetting

**Figure 5:**
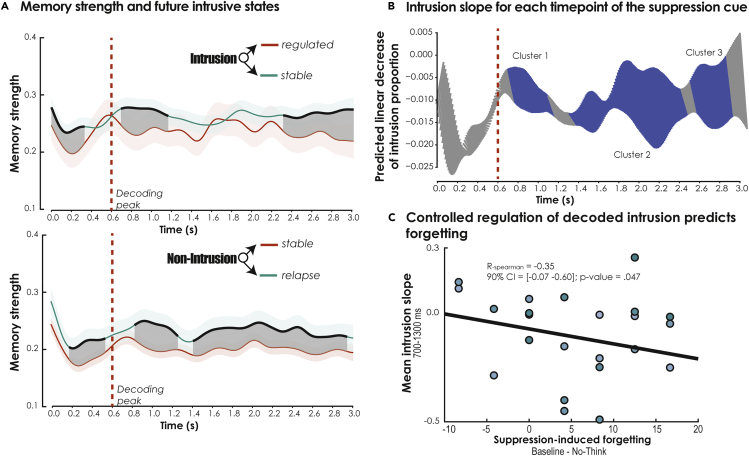
Temporal dynamics of intrusion control (A) Estimated marginal mean for intrusive memory strength according to future intrusive states. [top panel] Difference between regulated intrusive memories (i.e., intrusion in block N and non-intrusion in following blocks) and stable intrusive memories (i.e., intrusion in block N and in one of the following blocks) according to their future states. [bottom panel] Difference in intrusive memory strength for non-intrusive cues that remained stable (i.e., non-intrusion in block N and non-intrusion in following blocks) and ones that relapsed (i.e., non-intrusion in block N and in one of the following blocks) according to their future states. The dark area represents the significant differences, corrected for multiple comparisons across timepoints (p-corrected < 0.05). The dashed red line represents the decoding peak of the attentional model. Error bars represent 95% CIs. (B) For each timepoint of the suppression cue, we plotted the linear fit of the proportion of decoded intrusions (compared with non-intrusion) over the eight repetitions of the suppression task. The blue lines reflect the three temporal clusters showing the presence of a significant linear decline in decoded intrusions across participants, corrected for multiple comparisons across timepoints (p-corrected < 0.05). (C) Relationship between the intrusion slope (averaged within the significant time window detected after the decoding peak, dashed blue line, labeled *Cluster 1* in B) and suppression-induced forgetting.

We then wanted to understand how trials shift from intrusion to non-intrusion, and whether modulations in memory strength for a given presentation of a suppression cue predict the future control status of this trial during its subsequent presentations. We, therefore, split intrusive trials according to their future ratings during their remaining presentations. Accordingly, each intrusive item in a given TNT block was further qualified as either *stable intrusive memory* (i.e., intrusion in block *N* and in one of the remaining blocks) or *regulated intrusive memory* (i.e., intrusion in block *N* and non-intrusion in remaining blocks) according to its future state. It is important to note that this classification was made on an item basis (i.e., depending on the identity of the item). Any shift between *stable* and *regulated* intrusive memories would therefore reflect how control impacted the future intrusiveness of the memory trace and forgetting. We then compared the strength of mental event reactivation for these two distinct types of trials. Given that the numbers of both the trial presentations and the distinct items associated with these two conditions varied drastically between participants, we used a linear mixed effect (LME) model for this analysis, treating participants and item identity as random effects, and the future status of the memory (i.e., stable or regulated) as the fixed effect of interest. We found that a decrease in intrusive reactivation both just before and after the intrusion peak predicted the future absence of intrusion and the transformation of an intrusion to a non-intrusion. This downregulation of memory strength, predictive of the future intrusive status of the memory cue, occurred from 10 ms to 340 ms (*t* peak_(1229.3)_ = 2.69, cluster p value = 0.0375), 700 ms-1117 ms (*t* peak_(1182.3)_ = 2.86, cluster p value = 0.009), and 2310 ms-3000 ms (*t* peak_(1283)_ = 2.97, cluster p value = 0.0001), following stimulus onset (see Figure 5A, top panel). Interestingly, when we applied the exact same analysis to non-intrusive trials, dissociating non-intrusive cues that turned out to be stable from those that relapsed, we observed a very similar pattern to intrusive trials. Stable non-intrusive cues were associated with weaker memory than non-intrusive cues that relapsed, in both time windows that preceded (190 ms-550 ms, *t* peak_(353.2)_ = 2.93, cluster p value = 0.017) and followed (840-1270 ms, *t* peak_(843.18)_ = 3.45, cluster p value = 0.0035; 1420-2950 ms, *t* peak_(1223.8)_ = 2.87, cluster p value = 0.0005) the peak of decoding activity (Figure 5A, bottom panel). This finding revealed that the future intrusive state of suppressed memories can be detected using a model of reflexive orientating, even when attention is not initially oriented consciously. However, when the attentional capture of intrusive activity was detected consciously, early (i.e., <600 ms) and late (i.e., >600 ms) downregulation mechanisms increased the likelihood of intrusive memories being turned into silent traces.

Furthermore, memory adaptation induced by inhibitory control in the context of the memory suppression task was characterized by a decrease in intrusion proportion [*n*_intrusion_/(*n*_intrusion_ + *n*_non-intrusion_)] across TNT blocks (i.e., negative intrusion slope) and subsequent suppression-induced forgetting. The frequency of memory intrusions is known to gradually decrease across suppression sessions as participants exert inhibitory control - an effect associated with the magnitude of subsequent forgetting (Levy & Anderson, 2012). In this context, each trial is considered to be associated with a probability of intrusion equal to 1 or 0, depending on the participant’s subjective report. The sum of intrusive and non-intrusive trials is then used to compute the intrusion proportion for each repetition of the suppression cue, and to estimate the resulting slope using Pearson correlation coefficients. Here, we generalized this approach and computed a temporal intrusion slope using the binary decoding of intrusion across the timepoints of the suppression cue. We limited this approach to the timepoints that were correctly classified (i.e., true positive and true negative) and compared the resulting slopes with the baseline slope (i.e., −200 ms-0 ms) as a way of statistically comparing the significance of the slope against chance level. We found three significant clusters (cluster-based correction of p value below 0.05, 0.01, and 0.05, respectively; see Figure 5B) showing a significant negative slope, including an early cluster between 700 ms and 1100 ms (peak = 770 ms; *t* peak_(23)_ = −3.28, cluster p value <0.05), that immediately followed the time window associated with the maximum intrusive reactivation.

Previous studies had found that the steeper the intrusion slope, the greater the SIF. We sought to replicate this finding with our decoding approach, focusing our analysis on the significant post-intrusion cluster (i.e., Cluster 1 in Figure 5B). After averaging the intrusion slope within the post-intrusion time window, we found that participants who showed a steeper decline in decoded mental events immediately after the peak of intrusive activity exhibited more SIF (*R*_Spearman_ = −0.35; 90% CI = [-0.07, −0.60]; p = 0.047; see Figure 5C).

## Discussion

We used machine learning to track the transient intrusion of unwanted memories into awareness during a memory suppression task, using an algorithm trained on an early sensory and attentional interference signal triggered by the perception of an unexpected image in the visual field. We showed that this procedure generated significant predictions aligned with introspective reports of phenomenal awareness associated with intrusive memories. The overall performance of our model corroborates the notion that a relationship exists between the neurophysiological attentional and memory signatures of intrusive images. The comparison between a classifier involving the reflexive orienting of attention to the background image and a classifier that did not involve this bottom-up and automatic redirection of attention during the training of the algorithm, further underscored the role of attentional capture and reflexive attention during intrusive recollections. We also analyzed the strength and proportion of mental events decoded from brain activity to understand the temporal dynamics of intrusion regulation. For intrusive memories that penetrated conscious awareness, a reduction in the strength of detected mental events immediately preceding and following the peak of decoding activity (occurring on average 600 ms after the onset of the suppression cue) predicted the future regulation of these memories. Furthermore, when intrusive activity increased but was not consciously detected, a relapse in memory intrusiveness was observed in subsequent presentations of the same trial. Lastly, the proportion of mental events accurately decoded gradually decreased progressively with repeated suppression attempts - an effect was related to SIF. These findings suggest that our index of mental capture tracks not only the signal associated with the online reactivation of intrusive memories but also its regulation by inhibitory control to induce adaptive forgetting. Furthermore, the conscious detection of intrusive activity seems crucial to permitting the suppression of future intrusive states.

### Similarity between attentional and memory activities during intrusion

The emergence of unexpected visual activity into consciousness is often associated with the reflexive orienting of the internal focus of attention. To emulate this processing, we designed an object classification task involving the abrupt appearance of unexpected images presented in the background of the central target, thus disrupting its processing. Using random forest classifiers to decode the presence or absence of the unexpected image during this attention task, we found that maximum decoding accuracy peaked on average 110 ms after the presentation of the intrusive image (i.e., 310 ms after trial onset; see Figure 2A). This timing corroborated previous ERP and multivariate pattern analysis studies showing that early visual categorization occurs 100-200 ms after trial onset.^58^^,^^59^ Better decoding performance was also associated with positive components in the visual electrodes, and negative components in the prefrontal electrodes (see scalp representation in Figure 2A). This pattern may indicate the presence of early sensory processing intruding into perceptual awareness and capturing attentional resources. Compatible with this idea, using the same decoding procedure applied to time-frequency representations (see Figure 2B), we observed that the neurophysiological signature of early unexpected visual intrusion was dominated by frequencies centered in the theta range (3-7 Hz). Theta frequencies are engaged during attentional direction^25^^,^^60^ and memory interference detection.^61^^,^^62^^,^^63^^,^^64^

We then tested whether this model’s ability to decode the presence of unexpected visual intrusions could be extended to the detection of the involuntary recall of intrusive memories during the suppression task. This step relied on two assumptions: 1) intrusive memories take the form of visual memory activity that can be detected in EEG signals,^22^ and 2) the capture of internal attention triggered by intrusive memories shares neural properties with reflexive attentional processes allocated to external events.^24^^,^^26^^,^^65^^,^^66^^,^^67^ Confirming these assumptions, intrusive memories that transiently emerged during suppression cues in the TNT task were decoded above chance level by our attentional model. Multivariate decoding of EEG signals has been successfully applied to detect memory reactivation,^22^^,^^31^^,^^32^^,^^33^^,^^34^ different levels of conscious states,^68^^,^^69^ and mental imagery.^23^ Taken together, our findings suggest that theta activity, which dominated the early response to intrusive perceptual images, is central to the attentional capture of interfering activity that arises from both perception and memory. This conclusion fits with previous TNT studies showing that suppression is precisely associated with a reduction in theta activity.^38^^,^^48^^,^^49^^,^^70^

Moreover, our findings further revealed that mechanisms associated with perceptual activity and the voluntary direction of visual attention cannot account for intrusive memories as well as the neural mechanisms associated with the reflexive orientating of attention by sensory stimuli appearing unexpectedly in the peripheral field.^15^ This finding confirms that intrusive memories during memory suppression occur automatically in response to salient activity occurring outside the focus of attention, and show that reflexive attention and attentional capture are important for qualifying an intrusive activity as interfering and conscious. Our findings align well with theoretical models of consciousness suggesting a strong overlap between mechanisms of memory and visual awareness.^71^

Nonetheless, it should be emphasized that our decoding efforts did not capture the breaches in expectations induced by learning and memory-based predictions (i.e., prediction errors) that occurred during the TNT task. As recently demonstrated, these computational quantities can be powerful triggers of memory control processes,^72^ and memory-based prediction error is also probably a powerful signal that redirects the attentional system toward intrusion and triggers reactive inhibition. Our decoding approach only mimicked scenarios in which attentional redirection is purely contextual and not guided by a memory-based breach in expectation.

### Mental capture of intrusive events associated with inhibitory control induces adaptive forgetting

We also sought to analyze how repeated suppression attempts influenced the temporal profile of decoded events. Intrusive items whose intrusive memory strength was decreased immediately before or after the initial peak of intrusive activity (i.e., 600 ms) were more likely to be regulated during their subsequent presentations. These results corroborate observations that inhibitory control during memory suppression abolishes the item-specific EEG patterns associated with memory reinstatement.^50^ Moreover, non-intrusive cues that remained stable in subsequent suppression attempts were associated with a weaker memory strength than those that relapsed in subsequent blocks. These differences also occurred in time windows that both preceded and followed the peak of decoding activity. This finding reveals that the future intrusive state of suppressed memories can be detected from a model of reflexive orientation even when attention is not initially consciously directed. However, when the attentional capture of intrusive activity penetrates conscious awareness, downregulation mechanisms may increase the likelihood of turning intrusive memories into silent traces. Furthermore, the rate at which the proportion of decoded intrusions declined across the TNT repetitions, immediately after the transient detection of intrusive activity, was associated with later forgetting during the final retrieval phase, in line with previous observations based on behavioral reports.^4^

Taken together, these findings fit well with the proposed existence of inhibitory control mechanisms that regulate interfering memory activity both proactively and reactively.^72^^,^^73^ In the present study, the early regulation of mental events, presumably reflecting proactive control mechanisms engaged preventively before any recollection might happen to maintain task goals, was also significantly associated with the transitioning of intrusive memory to silent states (see Figure 5), in line with a recent report linking the early control mechanism to forgetting.^38^ It would be interesting for future studies to better characterize the relative contributions to the regulation of intrusive memories of predictive control mechanisms, grounded in the interaction between learning and control processes,^72^ and proactive control processes, grounded in the maintenance of task-relevant goals.^38^ This would enable them to better isolate the influence of these two distinct regulatory processes of conscious awareness on the memory engram. The modulation of intrusive activity observed after 600 ms is consistent with a reduction in the ERP correlate of recollection,^43^^,^^46^ in line with the proposed existence of a reactive control mechanism that disrupts the transient reactivation of intrusive memories by interrupting hippocampal processing.^72^^,^^73^ However, our findings further suggest that this interfering activity must not fall below the threshold of consciousness (i.e., labeled non-intrusion by participants) to be downregulated effectively and prevented from future relapse. The detrimental role of unconscious suppression (often associated with repression, but see^74^), as opposed to the beneficial role of conscious or intentional suppression in memory intrusiveness, has caused much debate in the literature.^74^^,^^75^^,^^76^ Our observations further suggest that beyond the level of consciousness associated with the act of inhibition itself, its potentially disruptive effect on the memory engram also depends on whether the memory activity targeted by suppression is conscious or not.

Moreover, given that we did not use an independent-probe test during the final recall test to ascertain the inhibitory nature of the SIF, decoded mental events may in fact reflect forms of competing interference that do require inhibition to disrupt the memory trace, as we suggest here.^77^ Although plausible, this non-inhibitory account of our findings seems quite unlikely, given that the experimental setting relied on a direct suppression instruction. Direct suppression instructions specifically ask participants not to retrieve distracting thoughts and to purge memories from awareness if they intrude. Participants are therefore effectively asked to shut down all retrieval in response to cues by blanking their minds. Some evidence suggests that direct suppression taps into inhibition. Compelling evidence for cue independence comes from studies using implicit memory tests, directly showing a suppression effect on the target memory, without probing its access with a reminder cue.^37^^,^^78^ The interference account predicts the presence of increased activity in the hippocampus (expressing interference), whereas hippocampal downregulation is commonly observed during direct suppression.^2^^,^^3^^,^^37^^,^^40^^,^^79^^,^^80^^,^^81^^,^^82^^,^^83^^,^^84^ There are several compelling arguments to suggest that this hippocampal downregulation is associated with inhibition. An amnesic shadow occurs after this downregulation.^39^ Meyer and Benoit (2022) also directly related the decline in vividness following suppression to a reduced reinstatement of unique memory representations in the right parahippocampal cortex (decoded from a one-back task that served to train a pattern classifier to detect evidence of scene reactivation). Hippocampal downregulation involves top-down negative coupling orchestrated by the prefrontal cortex,^2^^,^^3^^,^^10^^,^^37^^,^^72^^,^^80^ and is linked to the GABAergic inhibitory system.^40^ The inhibitory nature of the processes leading to forgetting is also subject to question, partly because the initiated memory response is not observable. However, common regions in the right dorsolateral and ventrolateral prefrontal cortex have been found to be involved when the target of inhibitory mechanisms is directly observable, such as in the motor domain, and when it is not, such as in the memory domain.^85^ Apšvalka and colleagues recently showed that stopping actions and thoughts recruits common prefrontal regions to suppress diverse content via dynamic targeting, supporting the existence of a domain-general system underlying inhibitory control.^86^ Finally, the findings on the temporal dynamics of intrusion control described above seem difficult to reconcile with a non-inhibitory interference account. In this theoretical framework, forgetting and intrusion regulation should be associated with the time window of maximum interference, during which its passive effect is at its height. Instead, we observed that the time periods following the peak of interference were more critical - a pattern more compatible with the presence of an inhibitory process engaged to countermand such interference. The above-mentioned evidence from the present study and previous ones suggests that the decoded events are memory-based, and their reactive regulation is supported by inhibitory processes, although we cannot exclude the possibility that non-inhibitory mechanisms may also contribute to some extent.

#### Implications for neurobiological models of forgetting

Our findings suggest that the regulation of interference activity signaling the unexpected emergence of intrusive memories into consciousness increases forgetting and a reduction in memory accessibility. A recent study suggested that this signal is detected by the dorsal anterior cingulate cortex, which then triggers prefrontal inhibitory control over hippocampal processing.^38^ Two mechanisms may explain why the involuntary recall of memory is a necessary condition for its forgetting. The first one assumes that the memory engram is disrupted during inhibitory control, while the second suggests that the suppressed memory trace is silenced, but may not be permanently forgotten.

First, memory recall has been hypothesised to engage consolidation mechanisms.^87^^,^^88^ Altering retrieval processes during intrusive memories may therefore alter consolidation mechanisms and the corresponding long-term potentiation of synapses, disrupting the excitatory connections that encode the memory engrams. Evidence indicates that suppression harms most active memory features via targeted inhibition.^13^^,^^37^ Partial reactivation of the memory trace associated with prediction error seems essential to trigger memory disruption,^89^ in line with the non-monotonic plasticity hypothesis whereby during memory reactivation, long-term depression of synaptic connections follows moderate postsynaptic depolarization.^12^^,^^90^ By reactively targeting intrusive reactivation, suppression may therefore maintain the activation of the memory trace at a moderate level of activity eligible for disruption.^12^^,^^90^ Another interesting, yet unexplored hypothesis, is that intrusive memory reactivation is coordinated by hippocampal sharp-wave ripples^91^ that normally permit reconsolidation.^88^ Disrupting sharp-wave ripples in reaction to intrusion may therefore also trigger a synaptic weakening of the suppressed memory trace.

Second, memory engrams may not only be composed of excitatory connections, but may also include inhibitory replicas, promoting the homeostatic regulation of postsynaptic excitatory currents.^41^ According to this model, memory recall is characterized by the disinhibition of these negative engrams, which reinstates neocortical activity in pyramidal cells that encode the bottom-up prediction error associated with the reactivated perceptual content.^92^ This neocortical disinhibition of the memory trace is orchestrated by the hippocampus, which amplifies the neocortical reinstatement of pyramidal neurons and regulates the precision of neocortical activity through attentional gain. In the present study, the mental capture induced by intrusive activity can be viewed as a form of prediction error. If the attentional gain of the prediction error orchestrated by the hippocampus is an essential mechanism for memory recall, its suppression may promote the plasticity of the negative engram, silencing the memory trace, which would nevertheless remain intact. Interestingly, this mechanism could explain recovered memories.^93^

### Conclusion

The electrophysiological signature of the interfering signal indicating a memory’s irrelevance or intrusiveness was found to be similar to the reflexive capture of attention by unexpected visual stimuli. We observed this signal online using machine learning and showed that its attenuation by inhibitory control induces forgetting. The role of intrusive memory in inducing inhibitory control and forgetting was observed here in the context of a memory suppression task, but the underlying attentional mechanisms discussed here may not be limited to this task, and may constitute a general mechanism that characterizes any task involving activity-dependent adaptive forgetting (e.g., directed forgetting, retrieval-induced forgetting). Future studies should be conducted with experimental paradigms other than the TNT task to assess the potentially critical and broader role of the attentional mechanisms discussed here. Retrieval-induced forgetting seems particularly relevant in this context, given that previous evidence indicates that it is linked to the suppression of the cortical reinstatement of interfering memories^14^ depending on theta oscillations^62^ and the causal involvement of the right prefrontal region thought to contribute to SIF.^94^

This finding has important implications for psychiatric disorders, such as posttraumatic stress disorder and obsessive-compulsive disorder, for which intrusive and interfering activities linked to memories or mental images are a central concern. These activities are often coupled with anticipatory behaviors, to avoid and prevent them in the first place, leaving the hippocampal-neocortical processing responsible for these intrusions intact. In future, the treatment of intrusive images might benefit from the development of attentional procedures that train mental operations to inhibit intrusive peripheral stimuli, paving the way for new treatments unrelated to problematic contents and promoting resilience.

### Limitations of the study

This study relied on a sample size of 24 participants, which can be considered small in the context of brain-behaviours correlation and limit the strength of the individual differences results. While a large number of trials allows for the estimation of precise individual parameters, the correlation between those parameters and the rate of forgetting would be estimated more reliably with a larger sample size. Moreover, our sample size does not allow us to detect small effect sizes, and most importantly, that the size of the effects reported here is likely overestimated compared with the true effect size that a larger sample will more accurately quantify.

The implicit underlying generative model of intrusive memories is constrained by the attentional task that was used to train the intrusion classifier. Such model could in theory vary, either regarding the electrophysiological signature of the intrusions, their temporal dynamics, or their probability of occurrence. Given that the ground truth of intrusive memory activities is unknown, a more systematic comparison of intrusion models and their fit to subjective reports would help to broaden the conjoint role of attention and memory expectations beyond the specific paradigm used in this study, and further generalize the predictive model of intrusive memories proposed here.

Another limitation of the present study is the absence of source localization of the attentional activity driving the decoding of intrusive memories which would help to clarify the nature of the mechanisms involved. Finally, we did not isolate the neural activity associated with inhibitory control and triggered in response to intrusive activity. Given that the central mechanisms associated with such inhibitory control of intrusive memories are manifested through the patterns of connectivity between the right dorsolateral prefrontal control regions and brain areas supporting the reactivation of memories, such goals would benefit from imaging approaches allowing the combination of high temporal resolution together with the study of brain connectivity, such as magnetoencephalography or simultaneous EEG-fMRI.

## STAR★Methods

### Key resources table

**Table:** 

REAGENT or RESOURCE	SOURCE	IDENTIFIER
**Deposited data**		

Behavioral and EEG decoded data	Zenodo repository	https://doi.org/10.5281/zenodo.7180660

**Software and algorithms**		

Python script for EEG processing and decoding	Zenodo repository	https://doi.org/10.5281/zenodo.7180660
MATLAB	https://mathworks.com/	version R2021b
PYTHON	https://www.python.org/	version 3.6

**Other**		

Bank of Standardized Stimuli	https://sites.google.com/site/bosstimuli/	version 2.0
Nencki Affective Picture System	https://lobi.nencki.gov.pl/research/8/	https://doi.org/10.3758/s13428-013-0379-1
Stimulus presentation	http://psychtoolbox.org/	Version 3.0

### Resource availability

#### Lead contact

Further information and requests for resources should be directed to and will be fulfilled by the lead contact, Pierre Gagnepain (pierre.gagnepain@inserm.fr).

#### Materials availability

This study did not generate new materials associated with this paper.

### Experimental model and subject details

A total of 27 participants (13 women) aged 18–35 years were paid to take part. Three of them were included to replace participants who did not report behavioural intrusions during the TNT phase. These three participants were not included in the group-level analyses, so 24 participants were analysed in total. The study was approved by the local ethics committee (CPP Nord Ouest I, no. ID RCB: 2015-A01727), and all participants gave their written informed consent. Participants were asked not to consume psychostimulants, drugs, alcohol, or coffee during the hours preceding the experimental period and to avoid intense physical activity the day before.

### Method details

#### Material

##### Attention

We selected 48 objects from the Bank of Standardised Stimuli (BOSS) (Brodeur et al., 2010, 2014) and 32 scenes from the Nencki Affective Picture System (NAPS) database.^95^ For the intrusion and exploration conditions, the stimuli were paired so that each object was associated with the same condition across the eight experimental blocks. The stimuli were divided into three subsets containing equal numbers of neutral and disgust stimuli (16 in each group). These three subsets were then assigned to the exploration, intrusion, and non-intrusion conditions (order counterbalanced across participants).

##### TNT

We selected 72 objects from the BOSS^96^^,^^97^ and 72 scenes from the NAPS.^95^ Six additional object-scene pairs were additionally selected for training purposes. All the items were different from those used in the attentional procedure. The stimuli were divided into three subsets containing equal numbers of neutral and disgust stimuli (24 in each group). These three subsets were then assigned to the Think, No-Think, and Baseline conditions (order counterbalanced across participants).

#### Procedure

##### Step 1 - Learning and test/feedback of TNT pairs

Participants learned the 72 object-scene pairs used in the TNT task plus the six training pairs. Participants first learned all the object-scene pairs in a test/feedback cycle procedure. After studying each of the pairs for 6 s, participants were given test trials presenting the object cue for a given pair for up to 4 s and asked whether they could recall and fully visualise the paired scene. If they did so, three scenes then appeared (one correct and two foils that were randomly taken from other pairs with the same emotional valence), and they had up to 4 s to select which scene went with the object cue. After selecting a scene, or if the response window expired, a screen appeared for 1 s indicating whether the recognition judgement was correct or incorrect. In all cases (even if participants indicated that they could fully visualise the associated scene in the first step), each trial ended with the correct pairing appearing onscreen for 2.5 s. Participants were asked to use this feedback to increase their knowledge of the pair. Once all the pairs had been tested, the total recall score (percentage) was displayed on the screen. If this percentage was less than 95% in at least one emotional condition, all the pairs were presented again in a randomised order. This procedure was repeated until the score was above 95%, thus ensuring the correct encoding of the pairs and comparable exposure to all the items.

##### Step 2 - Attention task

The attention task was intended to model and simulate memory intrusion during the TNT task, so we used a similar presentation paradigm. First, an image appeared in the centre of the screen, inside a red or green box. For red trials, participants were instructed to determine whether an object (non-living) or an animal (living) was presented and to provide answers as fast and as accurately as possible. Half the time, an interfering scene appeared in the background 200 ms after the onset of the object presentation (intrusion condition). For green trials, an image was displayed in the background 200 ms after the first stimulus presentation. Participants were instructed to determine whether humans (living) were represented or not (non-living) in this background image, and to provide answers as fast and as accurately as possible. Green boxes were associated with the exploration condition, and red boxes with the intrusion and non-intrusion conditions (no difference until the presentation of the intrusive picture on the screen). The larger picture only appeared in the background 200 ms after the cued-object presentation for intrusion and exploration trials.

The attention task was performed before the TNT task inside a Faraday cage. Participants were seated comfortably in a dimly lit room throughout the experiment, 90–100 cm from the screen, and were instructed to minimise blinking and moving during the recording. Stimulus presentation was controlled by E-prime Pro on a 17″ LCD screen with 1280 × 1024 resolution. To avoid exploratory eye movements, each picture was displayed inside a 400 × 400 pixel square. Intrusive and exploratory pictures were presented in the background with a dimension of 1024 × 768 pixels. All the images disappeared when participants responded, and their responses were followed by a jittered fixation cross randomly lasting 1300–1700 ms.

This task was divided into eight experimental blocks and lasted 27.05 min (*SD* = 3.76). Each block consisted of 16 exploration, 16 intrusion and 16 non-intrusion trials presented in random order. After two consecutive blocks, a message was displayed on a black screen telling participants that they could rest. The latter signalled when they wanted to resume the experiment by pressing any button on the response box.

It should be noted that participants were familiarized with the attention task before they entered the Faraday cage, by performing 12 trials (4 intrusion, 4 non-intrusion, and 4 exploration). The 12 objects and 8 scenes we used were completely new to participants and were not part of the TNT material or the attentional procedure material. This familiarization took place in front of a computer, and participants had to provide answers through the numerical keyboard: either 1 (living) or 2 (non-living). This order was consistent with the arrangement of the response buttons inside the Faraday cage.

##### Step 3 - TNT criterion test

At this point, participants viewed a brief reminder of all of the studied pairs (3 s each), during which they were asked once again to reinforce their knowledge of the pairings. Following this pair refresher, a final criterion test was administered, where participants had to recall the correct image in a similar way to the test/feedback procedure, but this time without feedback and only once. This allowed us to isolate items forgotten before the TNT task and exclude them from the analysis.

##### Step 4 - TNT task

All participants performed the task inside a Faraday cage after the attention task and criterion test. The TNT object cues were displayed inside a 400 × 400 pixel square. The green and red squares denoted the Think and No-Think conditions. The TNT task was divided into eight TNT blocks, each about 5 min in length. Each block consisted of the 24 Think and 24 No-Think items (12 neutral, 12 disgust), yielding 192 trials for No-Think and Think across the eight blocks. After two consecutive blocks, a message was displayed on a black screen telling participants they could rest. Participants signalled when they wanted to resume the experiment by pressing any button on the response box. It should be noted that although our design included both neutral and emotional material, we did not separate these conditions, as a drop in the number of total intrusions would have hindered the decoding analysis. Cues appeared for 3000 ms, framed in either green or red, centred on a grey background. In Think trials, the cue was inside a green box, and participants were told to generate an image of the associated scene that was as detailed and complete as possible. In No-Think trials, the cue was inside a red box, and participants were told that it was imperative to prevent the scene from coming to mind, and they should therefore fixate and concentrate on the object cue without looking away. During red-cued trials, participants were asked to block thoughts of the scene by blanking their mind, and not by replacing the scene with any other thoughts or mental images. If the object image came to mind anyway, they were asked to push it out. After the offset of each of the Think or No-Think trial cues, participants indicated whether the associated scene had entered awareness by pressing one of the response buttons. Three responses were possible: 1 (no intrusion at all, if the image did not intrude), 2 (the image intruded only for a short amount of time and/or was successfully controlled), and 3 (the image intruded vividly for a large amount of time). Participants had up to 2 s to choose their response, so they were instructed and trained to do so quickly, without thinking about the associated picture. Their response was followed by a jittered fixation cross randomly lasting 1300–1700 ms. These intrusion ratings were used to isolate trials with intrusive memories and quantify their occurrence. More specifically, we used participants’ responses to classify each trial as having an intrusion (i.e., 2 or 3) or not (1) in a binary fashion. This phase lasted 41.77 minutes (*SD* = 3.80 deviation). After two consecutive blocks, a message was displayed on a black screen telling participants they could rest. Participants signalled when they wanted to resume the experiment by pressing any button on the response box. After the first two blocks, the experimenter joined the participant in the Faraday cage to administer the last questionnaire concerning performance on the TNT task and ensure that the participant filled it out correctly. A *diagnostic* questionnaire was also used during the training and instruction for the TNT task, as well as before participants entered the Faraday cage.

##### Step 5 - Recall of TNT pairs

After this procedure, the after-effects of memory suppression were examined via a cued-recall task featuring all the object-scene pairs, including the 24 baseline scenes omitted from the TNT task. Because these items were trained at the same time as the Think and No-Think items but did not feature in the TNT task, they provided a baseline estimate of the memorability of the scenes, given that they were similarly old, but no suppression or retrieval had been performed on them. During trials of this cued-recall task, the cue object was displayed in the centre of the screen for 5 seconds, and participants were told to visually recall the associated scene. If they could do so, they were told to press the button under their right index finger. They then had up to 15 s to verbally describe the scene in as much detail as possible. Their descriptions were recorded. If they could not recall the associated scene, they had to press the button under their right middle finger, to trigger the display of the next object. Participants’ descriptions were checked by the first author, who was blind to the experimental conditions, to ensure that they corresponded to the relevant scenes and could not be confused with any others. On average, participants falsely recalled 1% of the images.

#### EEG recording

EEG activity was continuously recorded by a GES 300 amplifier (Electrical Geodesics, Inc. Eugene, OR, USA) using an EGI Hydrocel Geodesic Sensor Net (HGSN-128) with a dense array of 128 Ag/AgCl sensors. Impedances were kept under 100 kΩ, and EEG channels were referenced to a vertex reference Cz and ground to CPPZ (fixed by the EGI system). The signal was sampled at 20 kHz frequency with a 24-bit A/D and was online (hardware) amplified and low-pass filtered at 4 kHz. The signal was filtered by a Butterworth low-pass finite impulse response (FIR) filter at 500 Hz and subsampled at 1 kHz. An electro-oculogram was performed using four electrodes placed vertically and horizontally around the eyes. EEG data were processed offline using Netstation 4.4.2 (Electrical Geodesics Inc., Eugene, OR, USA). The signal was filtered using a 1 Hz Kaiser FIR first-order high-pass filter (which ensures a linear phase and no distortion in the bandwidth) to discard DC and very slow waves.

### Quantification and statistical analysis

#### Preprocessing

EEG analyses were performed using version 1.0.3 of the MNE library^101^^,^^102^ implemented in Python 3.6, and followed recently recommended analysis steps.^103^ Out of a total of 115 EEG electrodes, we removed 13 peripheral ones (F11, FT11, T9, TP11, P09, I1, Iz, I2, P010, TP12, T10, FT12, F12), owing either to poor impedance quality or lack of contact with the scalp. We low-pass filtered the raw data at a 30 Hz cutoff frequency using a FIR filter and referenced them to a common average. For the remaining 102 channels, sensors that showed no signal, constant white noise or an intermittent signal, as observed during the EEG recording, were interpolated. Cue onset was adjusted for the delay (+15 ms) between trigger generation and image appearance on the screen in the Faraday cage, measured using photodiodes during preliminary tests. We then segmented the trials from −1500ms to 2500ms for the attention task, and from −1500ms to 4000ms around the cue onset for the TNT task. We detected and corrected artefacts using the Autoreject library^104^ with local threshold detection and the Bayesian optimization method. Finally, we applied ICA correction to reduce the remaining eye blink artefacts, based on one manually selected EOG channel. The decoding analyses used time bins of 10 ms for the raw EEG signal, and 20 ms for the frequency signal.

#### Multivariate decoding

We used random forest classifiers as implemented in Scikit-learn v1.0.2^98^ to decode the appearance of an unexpected image on the screen during the attention task and to infer the presence of memory intrusion during the No-Think trials of the TNT task. A random forest algorithm is an ensemble method that combines many decision trees in a single model^99^. Each of the trees is built on bootstrap samples of the training data, and each split within a particular tree is made upon a random subset of the predictors. The advantages are that random forests can handle very high dimensional datasets, even when they contain a small number of observations or highly correlated predictor variables, and are less likely to overfit. As the number of trials could differ between conditions after preprocessing, we specified an unbalanced class weight parameter for our analysis and used 50 estimators. Data were normalised using the StandardScaller() function. All the other parameters were set at default values.

##### Decoding intrusion versus non-intrusion in the attention task

We used the preprocessed EEG recording from the 102 selected electrodes with 10-ms time bins. As some trials were excluded, based on Autoreject artefact detection^104^, and trimmed for aberrant reaction times (>3000 ms and <400 ms), the dataset contained different numbers of trials for each class. Although our design incorporated both neutral and disgusting intrusive images in the attentional and TNT procedures, we combined these stimuli here to improve the performance of the multivariate decoders. To control for overfitting and ensure that the classifiers were tested on independent data, the classification was evaluated through an eightfold cross-validation framework: each classifier was trained on 7/8 of the data and tested on the remaining 1/8. The performances of the eight predictors, indexed by the AUC, were then averaged and compared with chance level (0.5) for each timepoint, using a permutation-based cluster one-sample *t-*test that took the temporal distribution of the data into account. This allowed us to control for multiple testing and took the time dependence of the data into account.

##### Decoding intrusion versus non-intrusion in the attention task using time-frequency representations

To better understand the frequency dynamics of detecting and controlling unexpected images during the attention task, we repeated this approach using the time-frequency representation extracted using 27 Morlet wavelets from 3 to 30 Hz. This time, the input signal was the frequency power (3 to 30 Hz) at each timepoint (−500 ms to 1500 ms after the central image presentation, with 20-ms time bins). We corrected for baseline by computing the percentage of change compared with an interval ranging from −500 ms to 0 ms (percent method). The resulting time-frequency power was then downsampled to 50 Hz before further analysis. The classification was performed using the same decoding parameters as for the previous step, so only the input signal was different. The performance of the decoders was assessed using an eightfold cross-validation approach, and the performances of the eight predictors (AUC) were averaged for each participant. These performances were then compared with chance level (0.5), using a permutation-based cluster one-sample *t-*test, taking into account both the temporal and frequency distributions of the data. This time-frequency decoding approach was only used for the attention task as a confirmatory step.

##### Decoding intrusion versus non-intrusion during the TNT task, using the attention task

To decode memory intrusion during the TNT task, we relied on the temporal generalization method^55^ to infer the occurrence of similar intrusive patterns during this procedure, as learned from the attention task. First, we trained decoders to detect the presence of an unexpected image during the attention task. This part was performed using the SlidingEstimator() function in MNE Python. The predictors at each timepoint either side (−250 ms to 1500 ms) of the central image presentation were the raw EEG signal from the 102 selected electrodes after preprocessing, with 10-ms bins. To restrict our approach to intrusion-related activity, we only used the raw EEG signal 250–500 ms after the onset of the central object presentation (i.e., 50–300 ms after the presentation of the intrusive image in the background). This procedure ensured that our model captured the neural activity associated with the highest decoding accuracy of the abrupt intrusion of an unexpected image into conscious awareness identified during the initial attentional decoding. Using the same parameter, we then trained random forest decoders to classify intrusion versus non-intrusion trials from the TNT task. Using these decoders, we computed the probability associated with the intrusion condition for every timepoint of the No-Think trials on either side (−200 ms to 3000 ms) of the reminder presentation. This measure ranged from 0 (low likelihood of the given timepoint belonging to the intrusion condition) to 1 (high likelihood of the timepoint belonging to the intrusion condition).

For each participant and suppression trial, the classifiers provided a probability of intrusion for every timepoint of the suppression cue during the TNT task, as well as every timepoint of the attention task used for training, producing an *N*_attention_ x *N*_TNT_ timepoints matrix of probability. To further increase the reliability of our decoding procedure, we estimated the null distribution of intrusion probability by repeating the classification analysis 200 times with randomly permuted labels in the training dataset, as described by Linde-Domingo and colleagues (2019). We then used a threshold corresponding to the 95^th^ percentile of this null distribution to accept the prediction of an intrusion. This additional step reduced the odds of the prediction of an intrusive image being induced by noise in the EEG signal. The resulting binary map (corresponding to *N*_attention_ x *N*_TNT_ timepoints) for a given trial was then convolved with a Gaussian kernel (with a full width at half maximum of 200 ms), producing a timecourse of mental event reactivation describing the probability of an intrusion across No-Think trials. We then used this probability to infer the presence or absence of an intrusion. The label of each trial was based on the subjective report provided by the participant after a suppression cue. For each timepoint in the *N*_attention_ x *N*_TNT_ matrix, we then computed the resulting AUC using the TNT subjective reports as labels, and the probability of an intrusion across trials for this particular timepoint as a vector of classifier predictions. For each participant, we selected the attentional timepoint with the maximum AUC, producing a timecourse of the model classification performance across timepoints of the suppression cue. For subsequent analyses performed on the strength and proportion of mental intrusion (see “temporal dynamics of intrusion control” section), we used the timecourse of mental event reactivation and associated binary map that corresponded to the attentional timepoint with the maximum AUC.

We repeated this analysis twice according to two decoding scenarios. For the attentional model, we trained the decoding algorithm using the EEG signals from the intrusion and non-intrusion conditions. For the perceptual model, we applied exactly the same procedure, but using the EEG signals from the exploration and non-intrusion conditions.

#### EEG and decoding statistics

Statistical analyses of the behavioural data were performed using SciPy^105^ and Pingouin v.0.5.0 packages.^100^ EEG and decoding data were analysed using the MNE library v1.0.3^101^^,^^102^ and MATLAB R2021b. Standard paired or one-sample *t-*tests were used to estimate the significance of the decoding (i.e., AUC in time or time-frequency domain; Figure 2), memory strength (Figure 4), or intrusion slope timecourses (Figure 5). For the transition analysis (Figure 5), LME models were performed using the fitlme function of MATLAB, treating participants and item identity as random effects, and the future status of the memory (i.e., *stable* or *regulated*) as the fixed effect of interest. Parameters were estimated using the restricted maximum likelihood fit method and degrees of freedom were corrected using the Satterthwaite method. We controlled for possible false positives arising from the large number of comparisons in time-frequency and spatial plots using nonparametric statistical testing^57^ For each of these analyses, the initial cluster-forming threshold was set to a *p* value of 0.05. The *t* values of each sample inside the resulting clusters were then summed and compared with the maximum cluster statistics obtained after random permutations. The comparison between this *t-*value and the random permutation distribution provided a *p-*value that was reported and interpreted as the cluster *p-*value. 20000 permutations were performed for paired and one-sample *t-*tests, and 1000 for LME, as this analysis was computationally expensive. For the attention-to-TNT decoding, we kept the time windows of interest to 200–3000 ms, to limit the number of timepoints, but ensured that no significant differences before 200 ms were found in any of our analyses.

### Additional resources

None to declare.

## Data Availability

• All the behavioural data and the decoded EEG data have been deposited at Zenodo and are publicly available as of the date of publication. • All original code has been deposited at Zenodo and is publicly available as of the date of publication. DOIs are listed in the key resources table.
